# Running on a high: parkrun and personal well-being

**DOI:** 10.1186/s12889-017-4620-1

**Published:** 2017-07-25

**Authors:** Anne Grunseit, Justin Richards, Dafna Merom

**Affiliations:** 1 Prevention Research Collaboration, Sydney School of Public Health, University of Sydney, Level 6, The Hub, Charles Perkins Centre (D17), Sydney, NSW 2006 Australia; 2 Physical Activity and Health, Western Sydney University, Locked Bag 1797, Penrith, NSW 2751 Australia

**Keywords:** Physical activity, Mental health, Aging, Gender, Community event

## Abstract

**Background:**

Sporting or physical recreation event participation can affect different domains of mental and social well-being if sufficiently frequent, yet previous research has focused mainly on the physical health benefits of single-location or infrequent mass-participation events. We examined overall and domain specific subjective well-being of adult participants of “parkrun”, a weekly, community-based, highly accessible and widespread running event.

**Methods:**

Data were from a national online survey of 865 adult Australian parkrunners. Scores on nine individual measures and the global Personal Well-being Index (PWI) were compared to national, normative data. Regression models tested associations between personal well-being and perceived benefits of parkrun (mental health and connection to community).

**Results:**

Of 100 scores, 28% of means for parkrunners fell outside overall and age and gender subgroups normative ranges. *Satisfaction with health* was higher for male, those aged over 45 and overall parkrunners; only parkrunners aged 18–24 fell below their age group norm. S*atisfaction with life as a whole* was positively associated with perceived mental health benefits of parkrun, but not perceived community connection for women, and neither measure for men. PWI was positively associated with perceived community connection for men and with mental health benefit for women.

**Conclusions:**

Australian parkrunners mostly reflect the general population on personal well-being, except report superior satisfaction with physical health. Women’s personal well-being may benefit from parkrun through improved mental health and men’s from community connectedness. parkrun may facilitate positive expression of identity and continuation of healthy habits among athletes, and non-demanding, health enhancing activity and social interaction for non-athletes.

**Electronic supplementary material:**

The online version of this article (doi:10.1186/s12889-017-4620-1) contains supplementary material, which is available to authorized users.

## Background

Currently in Australia, approximately 60% of adults and two-thirds of children are not meeting the minimum physical activity guidelines [1]. Common barriers cited as preventing regular leisure-time physical activity are lack of time, low motivation, absence of skills, limited access to facilities, low social support and poor physical and/or mental health [2]. At the same time, the potential physical and mental health benefits are often given as the reasons for participation in health promotion events [3, 4]. While some community-based sporting events have been examined for their impact on population physical activity participation, these have generally been large scale but infrequent events (such as fun runs of varying lengths), or competitive national sport events involving serious athletes like the Senior Games in the USA [5]. It has been argued that such one-off events do not subsequently translate to regular uptake of physical activity among the general population [6]. Although there is some evidence in young people suggesting that voluntary sport programs in low-income settings attract motivated participants who are already the most active and fit [7] the assumption that this also holds true for large scale mass participation sporting events for adults is less well substantiated [8]. Further, much of the work within the public health discipline has focused on the expected benefits to physical health outcomes associated with increased participation in sporting or physical recreation events, with only limited attention paid to mental and social well-being [9].

Subjective well-being has been summarised as how people broadly evaluate their own lives [10]. The construct, it has been argued, includes not only a cognitive component pertaining to satisfaction with a range of life domains but also an affective component tapping people’s assessments of their happiness with life [11]. A range of measures of subjective well-being have been developed by researchers across various scientific disciplines and include global measures (e.g., overall life satisfaction), appraisals of particular domains such as relationships and experience of positive and negative affect. The significance of subjective well-being as a researchable concept is in its reflection of the quality of life within populations and subpopulations as well as a response to events, life situations and health promotion activities such as exercise or physical activity programs [12].

Cummins [13] in his development and use of the Personal Well-being Index (PWI) focuses on well-being as a subjective indicator of satisfaction with life as exemplified by ratings of “satisfaction with life as a whole”. He argues subjective well-being is actively managed and has a positive bias centring in the “satisfied” end of the spectrum. Other, more specific domains of well-being such as satisfaction with health or feeling part of the community, whilst when combined may approximate the more abstract “life as a whole” measure, demonstrate singly greater sensitivity to current events within that domain [13]. Testing has shown that although the index has remained stable (to within 3.1 points (range 0–100) over 13 years [14]), it still is sensitive to differences by gender, age and geographic location [15].

Review level evidence of observational studies has previously indicated that adolescents participating in sport score higher on psychological and social domains of well-being [16]. More recently, Kuykendall et al. [17] conducted a systematic review and meta-analysis of observational and experimental studies in adults and found that leisure engagement, including sport or active recreation, was consistently and strongly associated with subjective well-being. This was mediated by satisfaction from leisure and it appears that frequency of leisure-time activities is more important than total time of engagement. Importantly, the association between satisfaction from leisure and subjective wellbeing was moderated by age and country; it was stronger for retired individuals than those at the working age and also more pronounced in the European sample than the USA sample. These findings were attributed to differences in the cultural value assigned to leisure versus work according to age and geographical location. Similarly, gender has also been identified as an important moderating factor for the association between physical activity and indicators of subjective well-being, which may be due to differences in motivating factors and perceived benefits [9]. It is important to take into account demographic variation in measures of well-being and the interventions designed to increase it such that the latter can be appropriately targeted and tailored to maximise impact across the population.

Kuykendall et al. [17] also addressed the question of causal direction and demonstrated that increasing leisure activity of any type has a strong positive effect on subjective well-being. Further evidence of a causal relationship specific to a jogging/running event as the selected type of physical activity was identified in Sato et al.’s (2015) longitudinal study of distance running and life satisfaction. Specifically, participation in a distance running event is associated with a short term (one day post-event) increase in life satisfaction even in experienced runners with prior higher-than-average satisfaction, with subsequent diminution up to four months post-event [18]. The authors tested the mediating effect of psychological involvement (in running and the event itself) on life satisfaction, lending empirical evidence for this mechanism. However, the authors acknowledge the self-selected nature of the sample, a problem noted by others [8] and hence effects may be related to the propensity to participate rather than participation itself. Further, others argue that the true effectiveness of exercise and physical activity in promoting better health is yet to be properly estimated caught between the limited generalizability of efficacy trials and the selection bias of observational studies [19]. However, taken together, this evidence suggests that perhaps sporting events may have the potential to sustainably affect different domains of well-being providing they are sufficiently frequent.

The community-based sporting event “parkrun” (www.parkrun.com) is a free, five kilometre run staged on a weekly basis in 14 countries. Participants register online and obtain a personal barcode which is matched with a time and finishing place each time they participate in a parkrun anywhere globally. No minimum skill level or running ability are required. Results are forwarded via email to registrants shortly after each event showing their run time, placing overall and within their age group. Although parkrun is held in multiple locations globally (*N* = 1100) it is centrally organised with coordination at the regional, state, national and international levels. In Australia, at the time of writing there were 319,053 people registered across 213 parkrun sites and the average number of runs was 10 per runner [20]. Therefore, because it is regularly staged, community-based, readily accessible and comparatively widespread, parkrun represents quite a different format than the one-off annual “fun runs” that form the basis of most existing evidence about the benefits of mass-participation sporting events.

A study conducted with over 7000 parkrunners in the United Kingdom (UK) showed that the majority of parkrunners are not regular runners prior to registering for parkrun [21] and reported benefits for psychological well-being and sense of community, especially among more regular attendees. In a qualitative study of UK parkrunners, those interviewed referred to a number of features of parkrun that contributed to their initial and sustained engagement with the event such as social encounters, sense of achievement, reciprocity, opportunity for family participation, and building confidence [22]. Hence we may expect there to be a relationship between the perceived benefits of parkrun and participants’ sense of well-being. Apart from the studies conducted previously with parkrunners in the UK, however there is no other published research on the relationship of this event to participants’ well-being. Given the established influence of gender and age on subjective well-being [12] and its association with physical activity [9, 17], these demographic variables are important factors to take into account such that variation in response across sub-groups is made explicit and implications for interventions may be made clear.

Hence, the aims of this project were: i) to compare overall and domain specific subjective well-being of adult parkrun participants to the general population; ii) to identify which of the perceived physical, mental and/or social benefits of parkrun were associated with overall subjective well-being in adult participants, and iii) to investigate whether the associations differ by gender and age-group.

## Methods

### Procedure

In March 2015 one of parkrun’s Australian sponsors Stockland included an introductory paragraph and a hyperlink to a short online survey in the weekly newsletter sent to parkrun registrants (http://www.parkrun.com.au/news/2015/03/). The survey was open to anyone wishing to participate and the link was live for four weeks. No identifying information was collected and the data could not be linked to parkrun registrants’ administrative or run data. The authors obtained permission from Stockland and the parkrun Research Board to conduct a secondary analysis of these data. Ethical approval for the analysis was also granted by the University of Sydney Human Research Ethics Committee (approval #: 2015/932). Administrative data were also obtained from parkrun detailing the number of Australian registrants for each age category and gender at every parkrun event at the time of the survey (March 2015) for comparison purposes.

### Population and participants

The sample was drawn from parkrun registrants receiving the weekly newsletter and following the link to the survey. A total of 875 people submitted a survey. We excluded 10 respondents aged less than 18 years from the analysis [23], leaving an analytic sample size of 865.

### Survey instrument

The online survey questionnaire included questions which covered 1) demographic information: age (8 categories: 17 or under, 18–24, 25–34, 35–44, 45–49, 50–54, 55–64, 65 or older), Australian state of residence, gender, marital status, whether they live with children; 2) parkrun participation: whether participated in a parkrun in the last six months, 3) Personal well-being (described below); and 4) perceived benefits of parkrun (described below).

#### Personal well-being index

The Personal Well-being Index (PWI) comprises a series of seven statements, preceded by the stem “Thinking about your personal well-being, how satisfied or dissatisfied are you with the following?” The seven statements are: 1) *Your standard of living*, 2) *What you are currently achieving in life*, 3) *Your personal relationships*, 4) *How safe you feel*, 5) *Feeling part of your community*, 6) *Your health*, and 7) *Your future security*. Statements are rated on a 10-point scale ranging from 1 “completely dissatisfied”, through 5 “neither satisfied nor dissatisfied”, to 10 “completely satisfied”. For analysis and reporting, scores are multiplied by 10 and then a global index ranging from 0 to 100 is constructed by averaging scores over the seven statements [15]. Two additional questions using the same stem are rated on the same scale (*Your life as a whole*, *Your spirituality or religion*), but are not included in the global index [15]. The questions comprising the global index measure broad but identifiable and evaluable aspects of life that contribute to well-being. In combination with the more abstract “life as a whole” the scale and its components can reflect the immediate personal environment of the person, of which parkrun would be one factor. All nine individual items and the global scale have been separately validated as indicators of well-being and have been found to be stable and reliable [15].

#### Perceived benefits of parkrun

Three novel statements rated on a 5-point Likert scale (strongly disagree to strongly agree) measured participants feelings towards parkrun. Specifically: 1) *parkrun events help in increasing my physical health*, 2) *parkrun events help me in my mental health* and 3) *parkrun events help me connect with other community members*. Although are yet to be validated, these measures have face validity given the established perceived benefits of physical activity including releasing tension/improving psychological outlook and socialising [24, 25] and the acceptability of single-item measures especially for online surveys [26].

### Analysis

#### Factor structure testing and representativeness

Principal components analysis was conducted on the seven items comprising the PWI to check whether the single factor structure reported for the scale [15] was the same for the parkrun sample.

In order to determine whether the survey sample was representative of parkrun registrants, the age and gender profile of participants was compared to aggregate administrative data by chi-square.

As the analyses examining representativeness of the sample showed the survey sample differed significantly from the distribution for age and for gender of all Australian parkrun participants, sample weights were generated using the iterative proportional fitting procedure, as we only had access to marginal age and sex totals [27]. These weights were applied to the subsequent analyses.

#### Comparison of parkrun sample to Australian population normative range

The purpose of this component of the analysis was to examine whether parkrunners who are exposed to regular running events display similar overall and domain-specific well-being to their age and gender-specific general population counterparts, especially given previous research showing those who report running have higher well-being than the general population [28]. Results would be hypothesis-generating in that comparisons can show whether parkrunners are drawn from the general population or reflect a select group, irrespective of the direction of causality. The pattern over domains by age and gender can suggest how and to whom parkrun may be marketed. To this end, normative ranges for the PWI were calculated based on the 57,701 responses to the 33 cross-sectional studies that have used this metric from 2001 to 2016 in Australia. Whilst noting the stability of the measure of satisfaction with “life as a whole” and the global index mentioned above, the generation of norms based on numerous surveys over time contributes to the reliability of central tendency and variation. Original unit record data was supplied by the Australian Centre on Quality of Life (http://www.acqol.com.au/reports/auwbi.php). A normative range was created using recommended methods [29, 30]; specifically, means were calculated for each survey and the normative range calculated as +/−2 standard deviations from the mean of the 33 survey means overall, by gender, and by age group. The weighted (Table 1) and unweighted (Additional filel 1: Table S1) means for the current study were then compared with these ranges.

#### Perceived benefits of parkrun and subjective well-being

Spearman rank correlation between satisfaction with *life as a whole* and the PWI was 0.74, suggesting the two measures are related but not identical with the former being more abstract, and the latter reflecting more specific well-being domains combined [13]. Therefore, four linear regression models were used initially to examine the association between personal well-being (1. the single item satisfaction with *life as whole*, and 2. the global PWI) with perceived benefits of parkrun (1. mental health, and 2. connection to community) in bivariate models. Perceived benefit of parkrun to physical health was not included because of the lack of variation in response (97.6% agreed that parkrun helps with physical health). The association between perceived benefits and the two outcome measures were then tested in multiple variable models adjusted for age, gender, marital status, parkrun region by ARIA (major urban area, inner regional area, outer regional area) [31], and satisfaction with spirituality. The latter has been considered as a covariate due to the positive association between spirituality/religion and multiple indicators of health, in particular reduce risk of mortality [32]. The effect of spirituality has been attributed to the many “ingredients” it can provide that impact on wellbeing, including changes in life purpose, social support, coping mechanisms and resilience to stress [33]. Hence, to analyse the independent effect of the perceived benefits of parkrun we adjusted for the potential confounding effect of spirituality/religion on wellbeing. We analysed one further model with the single item *satisfaction with life as whole* adjusted for *satisfaction with your health* to examine whether this changed the effect of the two measures of perceived mental/social benefits of parkrun as health has been shown to be strongly related to overall well-being [34] and may mediate potential benefits of participation. Models were run separately for men and women because gender is a strong confounding factor for physical activity and may influence its association with subjective well-being [35]. Intraclass correlations (ICC) were calculated to test for clustering within parkrun.

All analyses were conducted using Stata 14.1 [36] using a significance threshold of 5%. Only those who had attended a parkrun in last six months (*n* = 850) were included in the analysis to ensure the sample were current parkrunners.

## Results

There were 155,189 parkrunners registered in the 96 parkruns from which survey respondents were drawn. Almost all survey respondents (97.2%) had participated in a parkrun event in the last six months.

### Representativeness of registered parkrunners

The comparison of gender distribution between the survey sample and parkrun registrants showed that the survey under-represented male parkrunners compared with registrants (38.5% vs. 42.6%, *p* = 0.016). With respect to age, the survey over-represented the three age groups 35–44, 45–49, 50–54 years compared with registrants (35.8% vs. 18.4%, 16.2% vs. 6.1%, 11.5% vs. 4.1%), and under-represented the three age groups 18–24, 55–64, 65 or older (2.4% vs. 6.3%; 10.8% vs. 35%; 3.7% vs. 10.3%) *p* < 0.001. For both the survey sample and parkrun registrants the 25–34 year-old group comprised 20%.

### Comparison of parkrun sample to Australian population on well-being

The PWI had a single factor structure which had a Cronbach’s alpha of 0.852 indicating high internal consistency.

The weighted means assessing each of the nine personal well-being items and the global PWI for the parkrun survey sample are shown in Table 1, with those falling outside the normative range for the population estimates bolded (normative ranges given in Additional file 2: Table S2); the cells with yellow fill are where our study sample had a mean score higher than the normative range (>mean + 2SD), and purple where our study sample had a mean score lower than the normative range (<mean – 2SD).

**Table 1:**
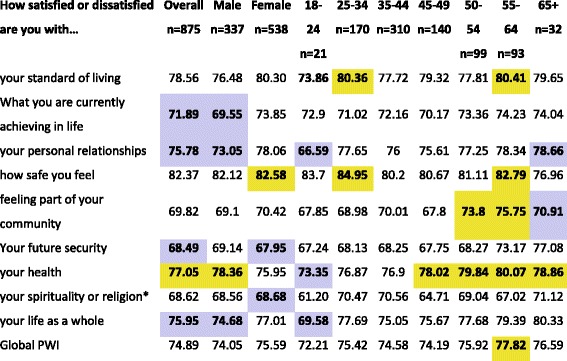
Sample means compared with normative ranges (*n* = 33 surveys) for Personal Well-being items and global index across age and sex

*Normative range based on 13 surveys

Yellow fill = survey sample mean score > mean + 2SD of reference group; mauve fill = survey sample mean score < mean – 2SD of reference group

Although the means for the survey sample fell mainly within the normative ranges for the majority of the measures, of the 100 scores 14 fell above and 14 fell below (28% in total). Patterns emerged across measures and subgroups for departures from the normative range: Personal satisfaction with health showed the most differences between parkrunners and the general population, with the sample as a whole, and the subgroups of males, and those aged above 45 years rating higher than the general population; only those aged 18–24 fell below the normative range for their age group (73.35 vs (74.6–85.1)). The global PWI showed only one departure from the normative range (55–64 years: 77.8 vs (73.7 to 77.0)). However, overall parkrunners, fell below the general population overall for *satisfaction with current achievement*, *personal relationships*, *future sec*urity and *life as a whole*.

Across the demographic subgroups, those aged 55–64 years rated themselves on average higher than the general population on half of the measures, including the PWI score; male respondents and those aged 18–24 were those who on average tended to rate their satisfaction lower than the general population on a range of measures. Sensitivity analyses without weighting showed differences from the weighted analyses in only a small number (*n* = 8, 8%) of cells with no systematic effect (lower or higher) of weighting.

### Regression analyses

ICCs for parkrun over the outcomes varied were very low (range < 0.001 to 0.018) and sensitivity analyses demonstrated no change to results when adjusting for clustering; therefore standard models are reported. The results of the bivariate linear regressions (Additional file 3: Table S3) showed that both *satisfaction with life as a whole* and the global PWI were significantly associated with perceived mental health benefits of parkrun (B = 4.08 & 3.93 respectively, *p* < 0.001) and community connection (B = 2.80 & 3.60, *p* = 0.004 & <0.001 respectively). There was a difference for perceived mental health benefit by gender (Additional file 3: Table S3); females (B = 4.70) scored significantly higher than males (B = 4.53, *p* < 0.001). However, there was no significant difference by gender for community connection, or for either measure by age group, therefore the multiple regression analyses were stratified by gender only, but included age as a covariate.

The results of the multiple linear regression for satisfaction with life as a whole and the PWI stratified by gender are shown in Table 2. Statistically significant results (coefficient and 95%CI) are italicised. The percentage variance accounted for by each of the four models ranged between 14.3% and 25.7%. For *satisfaction with life as a whole*, there was no significant association with either perceived mental health benefit or perceived community connection among male respondents. However, for women, the *satisfaction with life as a whole* score was significantly and positively associated with mental health benefits (*p* = 0.021), but not perceived community connection in the adjusted models (Table 2). When *satisfaction with your health* was added to the model, the results for mental health benefits stayed largely unchanged for men (B = 1.55, 95%CI: -1.61-4.70) but the association among women became non-significant (B = 2.45, 95%CI: -1.18-6.09) (data not shown).

**Table 2:** Adjusted effect of perceived mental health and community connection benefits of parkrun multiple regression for *satisfaction with life as a whole* and global PWI (*n* = 841) stratified by gender

	Satisfaction with life as a whole		Global well-being index (PWI)	
Perceived benefit	Male	Female	Male	Female
	B (95%CI)	B (95%CI)	B (95%CI)	B (95%CI)
Mental health	1.30 (−1.98–4.59)	*3.99 (0.60–7.38)*	0.37 (−2.35–3.10)	*3.76 (1.04–6.48)*
Community Connection	2.09 (−1.18–5.36)	0.04 (−2.39–2.46)	*3.60 (0.81–6.40)*	0.84 (−1.41–3.08)
	Rsq = 14.8%	Rsq = 14.3%	Rsq = 25.7%	Rsq = 22.5%

For the PWI, for men, there was a significant positive association between perceived community connection (*p* = 0.012), but not for mental health benefit. In contrast, for women, scores on the PWI improved significantly for perceived mental health benefit (*p* = 0.007), but not for perceived community connection. Other variables showing significant independent relationships across both outcome measures and men and women were marital status (being in couple versus being single increased life satisfaction/well-being scores by between 5.4 and 9.2 points) and *satisfaction with spirituality* (life satisfaction/global well-being scores increased between 2.1 and 2.4 points per 10-point increase in *satisfaction with spirituality*). Further, for women, scores for life satisfaction and global well-being scores were lower for those aged 35–49 years (compared with aged 50+ years) by 4.0 and 3.6 points respectively, and women attending a parkrun in an inner regional areas had on average scores on the PWI 4.8 points higher than those attending parkruns located in major urban areas.

Sensitivity analyses adjusting for area-level socioeconomic status (SES) by parkrun location as measured by the Socioeconomic Indexes For Areas (SEIFA) [37] showed minimal differences with the unadjusted analyses in terms of effect size, and no change in direction or statistical significance; a similar outcome (i.e., no notable change from the reported findings) was found for a supplementary analysis which included those who had not attended a parkrun event in the last six months.

## Discussion

To our knowledge our study is the first to explore established population indices of personal well-being in relation to a repeated mass participation community physical activity event and tested the extent the attributed perceived benefits, beyond physical health, are associated with well-being. Further, this is the first study of parkrun participants outside of the United Kingdom and only the second study worldwide, which has examined the parkrun population in relation to well-being. We found, firstly, that Australian parkrunners ranked higher than population norms on their perceived physical health, particularly older and male parkrunners. Secondly, when compared to the normative values, older parkrunners appeared to perform better than the general population across a number of well-being indicators, yet the youngest parkrunners (18–24 years) scored far below the age equivalent norms for the *health* and *personal relationships* sub-domains as well as for *satisfaction with life as a whole*. Thirdly, we identified sub-domains of well-being that were significantly lower than population norms among parkrunners which differed by gender; male parkrunners scored lower than the general population norms for their *current achievement in life*, *personal relationships* and *satisfaction with life as a whole*, whereas female parkrunners ranked very low on satisfaction with *future security* and *spirituality*. Fourthly, for men it appears that parkrun has the potential to improve overall personal well-being by facilitating social connections in the community, whereas for women it may be the mental health benefits from participating in parkrun that might enable improvements in well-being. Finally, there was an attenuation of the effect of perceived mental health benefit for satisfaction with life as a whole for women when *satisfaction with health* was taken into account perhaps signalling the close connection between the physical benefits of exercise and women’s mental health [38]. Each of these findings are discussed in detail below.

### Comparison of parkrunners vs. general population over well-being sub-domains

Satisfaction with health was higher among the parkrun sample as a whole, and most of the age and gender subpopulation groups, compared with national estimates based on the general population. This is consistent with previous research that shows satisfaction with physical health [39] and health-related quality of life [40] is higher amongst those who are physically active. Research from the UK shows that parkrunners represent a more active group when compared with the general population [21]. Given the well-established benefits of physical activity on health [41] it is therefore not surprising to find higher satisfaction with health among this group. However, the global measure of well-being, the PWI, was not higher than the normative range in the parkrun sample despite national analyses demonstrating that engaging in “strong” physical activity and/or exercise on five or more days per week are associated with PWI scores higher than the normative range [28]. The exception here was the 55–64 year subgroup who did fall above normative range, which is consistent with research showing that the benefit of exercise for well-being is more pronounced in older age groups [28]. Thus although the parkrun sample, potentially as a result of being physically active, were more satisfied with their health, their overall well-being appears to be at levels comparable with the general population. To reconcile these findings, we hypothesise that among our sample the positive effect of satisfaction with health on the overall PWI score was counteracted by lower scores for the other sub-domains (i.e., satisfaction with: *what currently achieving in life*, *personal relationships*, *future security*) also accounting for the different pattern of findings between the global index and the abstract single question *life as a whole*.

The consistency of scores at or above general population normative ranges for satisfaction with standard of living and safety may reflect the higher SES of parkrun participants in this sample (only 17% were attending parkruns which fell into the two lowest area-level SEIFA quintiles) and parkrun generally [21]. However, the absence of a cost-barrier makes parkrun ideal for lower income groups suggesting that perhaps there needs to be more effective engagement of these groups, especially given the SES gradient for meeting physical activity guidelines in Australia [1].

### Age

In terms of age, the youngest age group (18–24 years) showed scores which fell below the normative range for the general population on three sub-domains (*health*, *personal relationships* and *life as a whole*) and the oldest age group (aged 65+ years) on two sub-domains (*personal relationships* and *feeling part of community*). On the other hand, nine of the 11 instances where an age sub-population of the parkrun sample exceeded the normative range for their same-age counterparts in the general population occurred among those aged 45 years or older. This suggests that the parkrun participants who were the youngest, the oldest, and those aged 45–64 years were selective samples. For example, it is possible that parkrun appeals mostly to young participants who see this event as a way to improve their certain aspects of their health, (e.g., manage their weight). Indeed, research among UK parkrunners indicates that younger parkrunners are more likely to be former non- or occasional runners, groups which are more likely to be overweight and more frequently report physical (fitness, weight and health) and psychological benefits from parkrun than former regular runners [21]. Additionally, the trend for the general population is for the youngest groups to report the lowest satisfaction with personal relationships [28] and this was also the case for the parkrun sample. However, the number of parkrunners in the 18–24 years group was small (*n* = 21) and therefore the results should be interpreted with caution.

Lower satisfaction with *personal relationships* among the oldest age group (65+ years) may also reflect the specific circumstances of this period in the lifespan where loss of spouse is more common and retirement from work may contribute to reducing the quality and quantity of social connections [42]. As such parkrun may represent a good opportunity for the youngest and oldest age groups to improve their well-being by providing a regular and undemanding opportunity for positive social interaction simultaneous with health enhancing activity [22].

The parkrunners in the current study aged 45–64 years may also be a selective sample who are either attracted to the event to improve their physical health and fitness or because they were always athletic and still feel sufficiently competent to complete a five kilometre run [43]. Among UK parkrunners, 59% of the 55+ years age group were former regular runners compared with 39% of the 18–34 years group, which may suggest that older parkrunners are drawn from a more active population [21]. In the field of leisure studies these older participants are known as “serious leisure participants”, defined as the “systematic pursuit of an amateur, hobbyist, or volunteer activity sufficiently substantial and interesting for the participant to find a career there in the acquisition and expression of a combination of its special skills, knowledge, and experience” [44]. It is possible that older parkrunners have a similar profile to the older adult participants in the National Senior Games in the USA, a national athletic competition for individuals aged 50 years and older launched in 1987. Qualitative research with this group identified five central themes for participation: (a) perseverance, (b) career development and significant effort, (c) personal and social benefits, (d) unique ethos, and (e) identification as a senior athlete [45], which led authors to conclude that this event enhanced the well-being of older adults. For example, Senior Games participants developed an identity after the event of ‘senior athletes’ that was a central component of their daily life in terms of continuing training, improving their skills despite the physical decline and keeping track with other competitions. This suggested that self-identifying as an athlete contributes to high physical and cognitive functions that are major component of successful aging. Furthermore, these athletes developed a sense of social belonging and enhancement of self-image, which is another aspect of successful aging [5]. Whether parkrun older adults are serious active leisure participants can be determined by the regularity of their participation, but unfortunately one limitation of this study is that data on frequency of participation was not collected. However, as mentioned above, UK research shows the older age groups are likely to be regular runners prior to joining parkrun [21]. The lower normative scores in the oldest age group for *personal relationships* and *feeling part of their community* described above may be informed by different motivations whereby these participants use parkrun for casual leisure, which provides an “immediate rewarding, relatively short-lived pleasurable activity, requiring little or no special training to enjoy it” [44]. However, the cell size for this group was small (*n* = 32) and disproportionately low compared to the parkrun population of registrants.

Within the parkrun sample, female participants aged 35–49 had lower scores on both outcomes compared with those aged older than 50 years (only a three category variable was used due to small cell sizes) consistent with previous research that indicates scores tend to increase with age from approximately the age of 45 years in countries like Australia [34]. This trend is attenuated in people who experience major illness or health complaints, although did not appear to affect our sample of elderly parkrunners who scored above the population norms when considering satisfaction with their health.

### Gender

Women parkrunners reported higher satisfaction with how safe they feel compared with the general population. It is not clear whether this reflects higher participation by women who already feel safe, or that participation in parkrun has changed their perceptions about the safety of their communities. There is some evidence that the “perceived” rather than the “actual” safety of a community influences physical activity participation [46] and that safety may be a more important correlate of regular exercise than for men [47]. Either way, the nature of parkrun as taking place in large numbers and on terrain in parks or pathways which do not cross roads suggests that parkrun is likely to enhance feelings of safety, at least where it pertains to parkrun events in particular or physical activity in the local area more generally.

A significant association of perceived mental health benefits with both global well-being and *satisfaction with life as a whole* was only evident among female parkrunners in the stratified regression analyses; as the perceived mental health benefits of parkrun increased so did scores on these measures. Attenuation for this effect on the *satisfaction with life as a whole* scores if *satisfaction with physical health* is included in the model may suggest that the mental health benefit of parkrun is closely related to the physical benefits it confers. Research on correlates of physical activity has shown that health benefits of physical activity are salient for women [48] and therefore it may be that a proportion of the beneficial mental health effects of parkrun are mediated by improved physical health [38]. However, a prospective study design would be required to properly test this interpretation.

In contrast, opportunities for social interaction may be a more important mediator for achieving parkrun participation and well-being in men. The regression analyses showed that male parkrunners’ global well-being scores increased significantly as scores for perceived benefit of community connection increased. One study showed that across all three age groups of men (18–25, 35–45, and 50–65), social influences accounted for the most variation in moderate to vigorous physical activity. A qualitative study examining masculinity, sport and health emphasised the social dimension of sport as a major attraction for men [49]. Further, parkrun provides an opportunity for social connection through physical activity for men with a range of abilities without the pressure of performance for a sporting team, one of the detractions for some subgroups of men [49]. More generally, higher social support is associated with more frequent physical activity [50] and amongst parkrunners in the UK, higher sense of community was associated with more frequent parkrun participation [21] and instrumental to maintenance of attendance [22]. Together these findings suggest that the social nature and repeat format of parkrun may promote well-being through mutual reinforcement of positive social support and physical activity.

### Limitations

The current analysis had a number of limitations. Firstly the data were cross-sectional therefore the associations observed cannot be determined as causal. Future studies using longitudinal measures are indicated to clarify how parkrun and personal well-being interact. Although the sample size was reasonable, the confidence intervals for regression estimates were wide demonstrating low precision. It was also not possible to determine the response rate as there is no information available on the number of parkrun participants to whom the newsletter link was sent to or seen by. Given that respondents from 96 different parkruns across all Australian states and territories participated, however, there is some assurance that a range of parkrunners were reached. The distribution of the sample, as evidenced by comparison with registrant data, showed an age and sex distribution typical of voluntary surveys with under representation of the younger age groups and men [51]. Further, selection bias could also be affecting our results in that those who obtain the most benefit from parkrun may be more willing to complete a survey about this event than those who derive little or no benefit. Weighted and unweighted estimates for the normative ranges for the well-being measures were similar, however. Due to the survey being anonymous and therefore not able to be linked to participants’ parkrun data, the study did not include measures of frequency of attendance at parkrun which would allow an estimate of the dose-response effect of amount of exposure to parkrun on the variables of interest. Despite this limitation, our study demonstrates that parkrun may have the potential to impact established public health priorities and sets a platform for more rigorous research utilising a prospective evaluation design and linkage to running data routinely collected on parkrun participants. It also should be noted that the number of runners per event is a fraction of those registered and therefore the effects of parkrun discussed here are limited by the degree to which registrants become regular runners. Finally, the lack of association of between SES and the outcomes may be because we could only estimate the area-level SES of the sample from parkrun postcode as there was no SES information collected for individuals.

## Conclusion

Australian parkrunners in this study mostly reflected the Australian general population in terms of levels of personal well-being, with the exception of satisfaction with physical health where they are generally superior. Male and younger parkrunners fell below national normative ranges in some sub-domains where previous research has shown participation in parkrun may be beneficial. Future research could build on this and examine whether parkrun may offer support at critical times in the lifecourse where other sources of social connectedness and achievable physical activity may otherwise be lacking. Similarly, targeted promotion which highlights making connections within the community for men, and improved mental health mediated by better physical health for women, could capitalise on the gendered nature of perceived benefits of parkrun. As such, although causation is yet to demonstrated, parkrun may have potential to enable opportunities for the positive expression of identity and continuation of healthy habits among athletes, but also for non-demanding, health enhancing activity and social interaction for non-athletes.

## Additional files

Additional file 1: Table S1. Unweighted parkrun sample means for Personal Well-being items and global index across age and sex compared (*n* = 33 surveys) (DOCX 13 kb)

Additional file 2: Table S2. General population means and normative ranges (−2SD − +2SD) from *n* = 33 surveys (DOCX 15 kb)

Additional file 3: Table S3. Perceived mental health and community connection benefits of parkrun by gender and bivariate association with *satisfaction with life as a whole* and global PWI in overall sample (*n* = 841). (DOCX 12 kb)
