# Correction to: Running on a high: parkrun and personal well-being

**DOI:** 10.1186/s12889-019-7694-0

**Published:** 2019-10-16

**Authors:** Anne Grunseit, Justin Richards, Dafna Merom

**Affiliations:** 1 Prevention Research Collaboration, Sydney School of Public Health, University of Sydney, Level 6, The Hub, Charles Perkins Centre (D17), Sydney, NSW 2006 Australia; 2 Physical Activity and Health, Western Sydney University, Locked Bag 1797, Penrith, NSW 2751 Australia

**Correction to: BMC Public Health**

**https://doi.org/10.1186/s12889-017-4620-1**

It was highlighted that the original article [1] contained an error in the proportions reported in the Results, in the Representativeness of registered parkrunners section. This Correction article shows the incorrect and correct statement.

**Incorrect**:

With respect to age, the survey over-represented the three age groups 35–44, 45–49, 50–54 years compared with registrants (35.8% vs. 18.4%, 16.2% vs. 6.1%, 11.5% vs. 4.1%), and underrepresented the three age groups 18–24, 55–64, 65 or older (2.4% vs. 6.3%; 10.8% vs. 35%; 3.7% vs. 10.3%) *p* < 0.001. For both the survey sample and parkrun registrants the 25–34 year-old group comprised 20%.

**Correct**:

With respect to age, the survey over-represented the five age groups 35–44, 45–54, 55–64 years, compared with registrants (35.8% vs. 31.0%, 27.6% vs. 17.2%, 10.8% vs. 5.9%, 3.7% vs 1.7% respectively), and underrepresented the two age groups 18–24 and 25–34 years (2.4% vs. 10.7%; 19.7% vs. 33.6% respectively) *p* < 0.001.
