# Supplementary material for: Running on a high: parkrun and personal well-being
Source: BMC Public Health. 2017 Jul 25;18:59. doi: 10.1186/s12889-017-4620-1 (PMC5526231; doi:10.1186/s12889-017-4620-1)
Supplement: Supplementary file 1 — Unweighted parkrun sample means for Personal Well-being items and global index across age and sex compared (n = 33 surveys) (DOCX 13 kb) [file 12889_2017_4620_MOESM1_ESM.docx]

Table S1: Unweighted parkrun sample means for Personal Well-being items and global index across age and sex compared with general population normative ranges (n=33 surveys)

|  | **Overall** | **Male** | **Female** | **18-24** | **25-34** | **35-44** | **45-49** | **50-54** | **55-64** | **65+** |
| --- | --- | --- | --- | --- | --- | --- | --- | --- | --- | --- |
|  | **n=875** | **n=337** | **n=538** | **n=21** | **n=170** | **n=310** | **n=140** | **n=99** | **n=93** | **n=32** |
| **Standard of living** | 78.96 | 77.88 | 79.64 | 76.19 | **80.59** | 77.65 | 79.57 | 77.68 | **81.08** | 80.00 |
| **Currently achieving in life** | 72.42 | **70.45** | 73.66 | 74.29 | 71.53 | 72.26 | 70.36 | 74.04 | 74.84 | 74.38 |
| **Relationships** | **76.89** | **74.75** | 78.25 | **69.52** | 77.47 | 76.32 | 76.14 | 77.47 | 79.46 | **78.13** |
| **Safe you feel** | 81.45 | 81.82 | 81.21 | 84.76 | **84.94** | 79.94 | 80.64 | 80.81 | **82.69** | 77.19 |
| **Part of community** | 70.64 | 69.94 | 71.08 | 67.62 | 69.29 | 70.10 | 68.21 | **73.84** | **75.91** | **70.31** |
| **Future security** | 68.84 | 70.15 | **68.02** | 67.62 | 68.24 | 67.67 | 68.00 | 68.18 | 73.23 | 77.19 |
| **Your health** | **77.57** | **78.78** | **76.81** | **72.86** | 76.71 | 76.52 | **77.86** | **79.49** | **80.54** | **79.69** |
| **Spirituality or religion*** | 68.95 | 68.00 | **69.55** | 60.48 | 70.18 | 70.71 | 65.29 | 68.79 | 67.63 | 71.25 |
| **Life as a whole** | 76.69 | 75.73 | 77.30 | **70.48** | 77.76 | 75.16 | 76.07 | 77.68 | 79.78 | 80.63 |
| **Global PWI** | 75.28 | 74.91 | 75.52 | 73.27 | 75.54 | 74.44 | 74.40 | 75.93 | **78.25** | 76.70 |

* Based on 13 surveys as not asked in every survey

Yellow fill = survey sample mean score >mean + 2SD of reference group; mauve fill = survey sample mean score <mean – 2SD of reference group.
