# Supplementary material for: Running on a high: parkrun and personal well-being
Source: BMC Public Health. 2017 Jul 25;18:59. doi: 10.1186/s12889-017-4620-1 (PMC5526231; doi:10.1186/s12889-017-4620-1)
Supplement: Supplementary file 2 — General population means and normative ranges (−2SD − +2SD) from n = 33 surveys (DOCX 15 kb) [file 12889_2017_4620_MOESM2_ESM.docx]

Table S2: General population means and normative ranges (-2SD-+2SD) from n=33 surveys

|  | **Overall** | **Male** | **Female** | **18-24** | **25-34** | **35-44** | **45-49** | **50-54** | **55-64** | **65+** |
| --- | --- | --- | --- | --- | --- | --- | --- | --- | --- | --- |
| **Standard of living** | 78.09  (75.48-80.69) | 77.6  (74.77-80.43) | 78.53  (75.63-81.43) | 80.78  (75.72-85.85) | 76.44  (73.62-79.25) | 76.23  (72.65-79.82) | 76.07  (72.47-79.67) | 76.92  (73.68-80.16) | 78.38  (75.88-80.87) | 80.75  (77.87-83.64) |
| **Currently achieving in life** | 73.59  (71.97-75.21) | 72.63  (70.85-74.41) | 74.48  (72.49-76.48) | 73.63  (69.54-77.73) | 73.07  (70.44-75.7) | 72.91  (70.75-75.06) | 71.61  (67.95-75.27) | 72.22  (69.03-75.41) | 74  (71.07-76.93) | 75.79  (72.11-79.48) |
| **Relationships** | 79.54  (77.61-81.46) | 78.4  (75.99-80.81) | 80.56  (77.87-83.26) | 75.92  (71.05-80.78) | 79.23  (75.61-82.86) | 78.85  (75.94-81.75) | 77.64  (73.36-81.93) | 78.07  (74.02-82.11) | 79.99  (77.14-82.84) | 82.69  (79.28-86.11) |
| **Safe you feel** | 79.29  (75.77-82.8) | 79.99  (76.69-83.3) | 78.62  (74.72-82.53) | 81.39  (74.98-87.81) | 79.72  (75.3-84.15) | 79.72  (75.79-83.66) | 79.73  (75.66-83.8) | 79.45  (75.26-83.64) | 78.59  (74.51-82.67) | 78.78  (74.99-82.56) |
| **Part of community** | 71.25  (68.8-73.69) | 69.58  (66.99-72.17) | 72.81  (69.91-75.71) | 66.99  (61.71-72.27) | 67.23  (63.6-70.86) | 70.46  (67.07-73.84) | 70.48  (66.81-74.15) | 70.81  (68.41-73.21) | 72.12  (70.27-73.98) | 75.25  (72.72-77.78) |
| **Future security** | 71.23  (68.62-73.84) | 70.91  (68.18-73.64) | 71.52  (68.49-74.54) | 71.71  (66.92-76.5) | 69.57  (66.4-72.74) | 69.9  (66.36-73.45) | 68.77  (65.95-71.59) | 69.33  (66.15-72.52) | 71.06  (67.68-74.44) | 75.12  (71.83-78.41) |
| **Your health** | 74.55  (73-76.11) | 74.09  (72.23-75.95) | 74.97  (73.22-76.71) | 79.85  (74.6-85.1) | 77.38  (74.58-80.18) | 76.12  (73.74-78.5) | 74.71  (71.63-77.79) | 73.72  (71.52-75.92) | 72.78  (69.84-75.73) | 72.25  (69.22-75.27) |
| **Spirituality or religion*** | 73.78  (65.37-82.18) | 71.02  (60.44-81.61) | 76.26  (70.07-82.45) | 72.72  (60.4-85.05) | 73.56  (64.25-82.87) | 72.72  (63.34-82.1) | 72.73  (63.08-82.39) | 72.47  (63.31-81.63) | 73.83  (66.34-81.32) | 76.02  (69.21-82.83) |
| **Life as a whole** | 77.62  (76.1-79.13) | 76.89  (74.98-78.8) | 78.28  (76.5-80.06) | 76.48  (73.28-79.67) | 76.21  (73.91-78.5) | 76.21  (74.18-78.24) | 75.51  (72.67-78.34) | 75.97  (73.33-78.6) | 78.08  (75.64-80.52) | 81.03  (78.15-83.91) |
| **Global PWI** | 75.41  (73.79-77.04) | 74.81  (72.95-76.66) | 75.97  (74.09-77.84) | 75.83  (71.73-79.92) | 74.68  (72.67-76.69) | 74.96  (72.75-77.17) | 74.22  (71.41-77.03) | 74.4  (72.58-76.22) | 75.32  (73.67-76.97) | 77.31  (75.17-79.46) |

* Based on 13 surveys as not asked in every survey
