# Supplementary material for: Running on a high: parkrun and personal well-being
Source: BMC Public Health. 2017 Jul 25;18:59. doi: 10.1186/s12889-017-4620-1 (PMC5526231; doi:10.1186/s12889-017-4620-1)
Supplement: Supplementary file 3 — Perceived mental health and community connection benefits of parkrun by gender and bivariate association with satisfaction with life as a whole and global PWI in overall sample (n = 841). (DOCX 12 kb) [file 12889_2017_4620_MOESM3_ESM.docx]

Table S3: Perceived mental health and community connection benefits of parkrun by gender and bivariate association with *satisfaction with life as a whole* and global PWI in overall sample (n=841).

|  |  | |  | |  | | **Satisfaction with life**  **as a whole** | | | **Global well-being index (PWI)** |
| --- | --- | --- | --- | --- | --- | --- | --- | --- | --- | --- |
| **Perceived benefit** | Male  Mean | Female  Mean | | B (95%CI) | | p-value |  | B (95%CI) | B (95%CI) | |
| **Mental health** | 4.53 | 4.70 | | 0.17 (0.07-0.27) | | 0.001 |  | **4.08 (1.81-6.43)** | **3.93 (2.09-5.78)** | |
| **Community Connection** | 4.49 | 4.56 | | 0.08 (-0.44-0.20) | | 0.214 |  | **2.80 (0.88-4.72)** | **3.60 (1.99-5.20)** | |
